# Effect of Ni-Cr Alloy Surface Abrasive Blasting on Its Wettability by Liquid Ceramics

**DOI:** 10.3390/ma14082007

**Published:** 2021-04-16

**Authors:** Weronika Czepułkowska-Pawlak, Leszek Klimek, Marcin Makówka, Emilia Wołowiec-Korecka

**Affiliations:** Institute of Materials Science and Engineering, Faculty of Mechanical Engineering, Lodz University of Technology, Stefanowskiego 1/15, 90-924 Łódź, Poland; leszek.klimek@p.lodz.pl (L.K.); marcin.makowka@p.lodz.pl (M.M.); emilia.wolowiec-korecka@p.lodz.pl (E.W.-K.)

**Keywords:** wettability, Ni-Cr alloy, dental ceramics, abrasive blasting, alumina, silicon carbide

## Abstract

An adequate surface is essential in ensuring a solid bond between the metal and dental ceramics for metal framework wettability. This work is aimed at investigating the effect of variable abrasive blasting parameters on Ni-Cr alloy surface’s ability to be wetted with liquid ceramics at elevated temperatures. One-hundred and sixty-eight samples were divided into 12 groups (*n* = 14), which were sandblasted using variable parameters: type of abrasive (Al_2_O_3_ and SiC), the grain size of the abrasive (50, 110, and 250 µm), and processing pressure (400 and 600 kPa). After treatment, the samples were cleaned in an ultrasonic cleaner and dried under compressed air. Dental ceramics were applied to the prepared surfaces via drops, and the wettability was tested in a vacuum oven at temperatures in the range of 850–1000 °C. The results were statistically analyzed using ANOVA (α = 0.05). For all surfaces, the contact angles were less than 90° at temperatures below 875 °C. For Al_2_O_3_, the best wettability was observed for the smallest particles and, for SiC, the largest particles. The ability to wet the surface of a Ni-Cr alloy is related to its sandblasting properties, such as roughness or the percentage of embedded abrasive particles. It should not be the only factor determining the selection of abrasive blasting parameters when creating a prosthetic restoration.

## 1. Introduction

Metal–ceramic restorations in the form of crowns and bridges are widely used in dental prosthetics, where the ceramic material is fired onto a metal substructure. Such prostheses are characterized by their pleasing aesthetics and durability, reaching ten years of use [1,2,3,4]. Thanks to ceramics’ relatively smooth surface, plaque does not stick to its surface [5].

The metal–ceramic connection is widely tested because of differences between the properties of the materials [6,7,8,9]. In some cases, ceramics crack or chip from the metal surface [10,11,12,13]. Such damage is difficult to repair; therefore, a solution is sought to ensure the most durable connection between the materials.

There are several mechanisms in the connection between metal and ceramics. The first is to ensure that the ceramics are mechanically attached to irregularities in the metal surface. During firing, the semi-liquid ceramic flows into grooves that result from abrasive blasting of the alloy surface. This treatment’s parameters are fundamental because they affect the size of the created unevenness and may affect the joint’s durability and strength [14]. The use of abrasive particles that are too small or too large may cause insufficient surface roughness for the ceramic to attach well enough. Particles that are too small will cause the width and depth of unevenness to be too small, and ceramics with a low viscosity will not flow into them and will easily chip from the metal surface at a later stage. Particles that are too large will cause the width and depth of the unevenness to be too large, which may result in insufficient attaching [15]. Another mechanism that is said to be part of the metal–ceramic connection is the chemical bond. There are reports in the literature that diffusion of elements and the formation of chemical bonds between materials occurs at the metal–ceramic interface [9]. The last mechanism concerns the connection that provides the difference in the coefficients of thermal expansion (CTE) between the materials [8,9]. The difference in thermal shrinkage between the materials results in the creation of compressive stress in the ceramic during cooling, which increases the joint’s strength. The clamping of ceramics occurs, not only on the prosthetic element, but also on the unevenness. Their sizes and shapes are given by the abrasive blasting effect on the joint’s quality.

All the described mechanisms are components of the metal–ceramic connection. However, the mechanical attaching of ceramics is the most important mechanism. Appropriate metal surface treatment increases the surface and its influence on a restoration’s strength is visible [16]. Abrasive blasting of a metal affects the surface conditions, the parameters of which are roughness and wettability or the percentage of embedded abrasive particles [1]. Analysis of the wettability of the surface should be crucial in creating a restoration. However, dental ceramics change their characteristics from hydrophilic to hydrophobic under the influence increases in temperature. When it comes into contact with a metal surface at room temperature, the ceramic is a mixture of powder and water. As the temperature increases during firing, the water in the material evaporates, and the characteristic of the ceramic changes. Therefore, the study of surface wettability by measuring liquids gives incomplete information on its influence on the connection. The purpose of this study was to analyze the impact of various parameters of abrasive blasting on the wettability of Ni-Cr alloy surfaces by liquid ceramics at varying temperatures.

## 2. Materials and Methods

One-hundred and sixty-eight Heraenium^®^ NA nickel-chromium alloy samples (Heraeus Kulzer, Hanau, Germany) were obtained commercially as ready-made elements for prosthetic works, with cylindrical shapes with a diameter of 8 mm and height of 15 mm. The chemical composition is presented in Table 1. The alloy’s chemical composition was determined by the X-ray fluorescent analysis method using an SRS300 spectrometer (SIEMENS, Berlin, Germany). Samples were divided into two groups and were abrasion blasted (Alox 2001, Effegi Brega, Sarmato, Italy) using alumina (Al_2_O_3_) or silicon carbide (SiC) for 20 s, with a nozzle inclination of 45° and at a distance of 15 mm from the surface of the material. Every group of samples was divided into six subgroups (*n* = 14). The groups were distinguished by the abrasive blasting parameters where the abrasive particle size and processing pressure were the variables. Designations of the samples are presented in Table 2.

After abrasive blasting, all samples were cleaned in an ultrasonic cleaner (Emmi-55HC-Q, Emag, Mörfelden-Walldorf, Germany) with deionized water for eight minutes to remove loose abrasive particles. Then the surface was dried under compressed air.

Dental ceramics IPS Classic (Ivoclar Vivadent, Schaan, Lichtenstein) were dropped onto the prepared surfaces, and the surface wettability was tested in a tube furnace designed for this activity with the possibility of connecting a camera. Measurements were made every 25 °C in the temperature range 850–1000 °C based on sample photos, which were used to determine the values of contact angles by measuring the geometric features by drop shape analysis. Contact angles were calculated based on images made at different temperatures after various surface treatments. The method of measuring the angle was taken from the Śmielak et al. study [17]. The contact wetting angle was determined according to Young’s equation from the 3-phase contact point [17] for each analyzed temperature according to the formula:

(1) $$\left|\bar{\sigma_{LV}}\right|\times \cos\theta +\left|\bar{\sigma_{SL}}\right|-\left|\bar{\sigma_{SV}}\right|=0\;$$

where *θ*—contact angle, *σ_SV_*—surface tension at the solid-gas interface, *σ_SL_*—surface tension at the solid-liquid interface, *σ_LV_*—surface tension at the liquid–gas interface. The value of the contact angle is measured on both the left and right sides of the drop according to geometric parameters of specimens [17].

Additionally, the relative wetting force was calculated for the performed experiments by relating the wetting forces of individual groups to a reference group (Al_2_O_3_, 400 kPa, 110 µm abrasive). This group was selected as a reference group because these parameters of treatment are assumed to be the best for metal-ceramic connection in dentistry [14,18]. Wetting force *F_c_*, taking into account surface tension σ_LV_, the contact angle θ, and the circumference of the sample *O_p_*, results from the relationship:

(2) $$F_{c}=O_{p}\times \sigma_{LV}\times \cos\theta_{0}$$

Thus, it is possible for the extreme contact angles obtained in the previous experiments to determine the relative contact force as a relationship defined by the ratio of the contact angles:

(3) $$\frac{F_{2}}{F_{1}}=\frac{O\times \sigma_{LV}\times \cos\theta_{2}}{O\times \sigma_{LV}\times \cos\theta_{1}}=\frac{\cos\theta_{2}}{\cos\theta_{1}}$$

Statistical analyses of the results were conducted by using the Statistica statistical software. A 2-factor ANOVA and a post hoc Tukey’s test were conducted (α = 0.05).

## 3. Results

**Figure 1:**
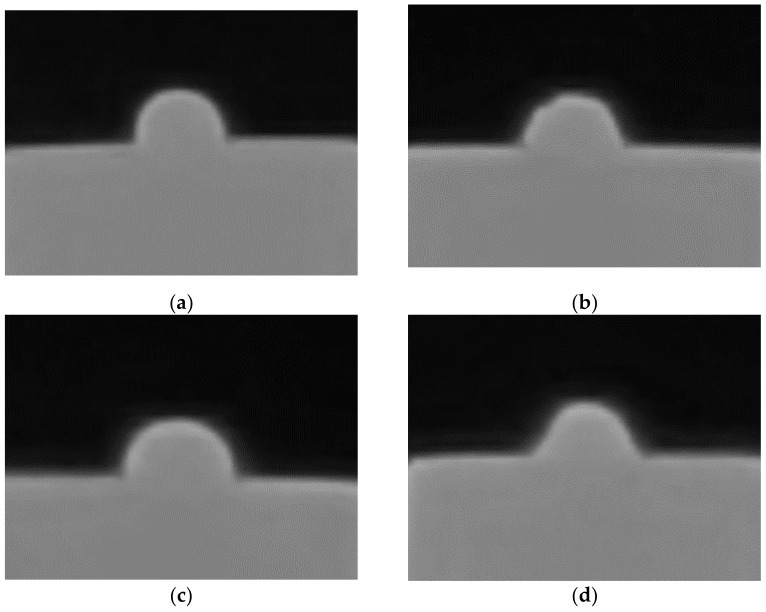
Images of a drop of ceramics on tested samples: (**a**) on the sample treated with Al_2_O_3_ (250 µm, 600 kPa) at 850 °C, (**b**) on the sample treated with Al_2_O_3_ (250 µm, 600 kPa) at 1000 °C, (**c**) on the sample treated with SiC (50 µm, 600 kPa) at 850 °C, (**d**) on the sample treated with SiC (50 µm, 600 kPa) at 1000 °C.

Figure 1 shows photos of a ceramic drop on alloy samples, which were used to determine the contact angles.

**Figure 2:**
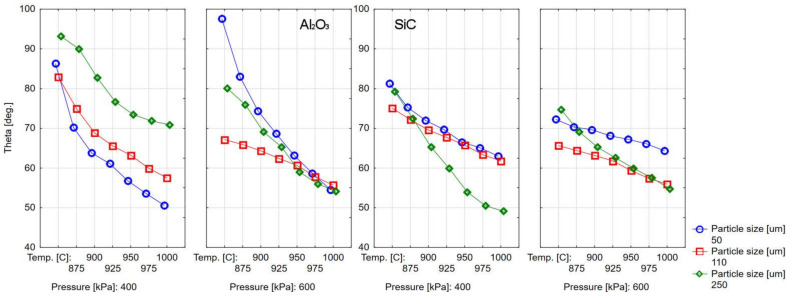
Graph of the dependence of the surface’s contact angles on the temperature and abrasive blasting parameters: type, size, and pressure of the abrasive particles.

**Figure 3:**
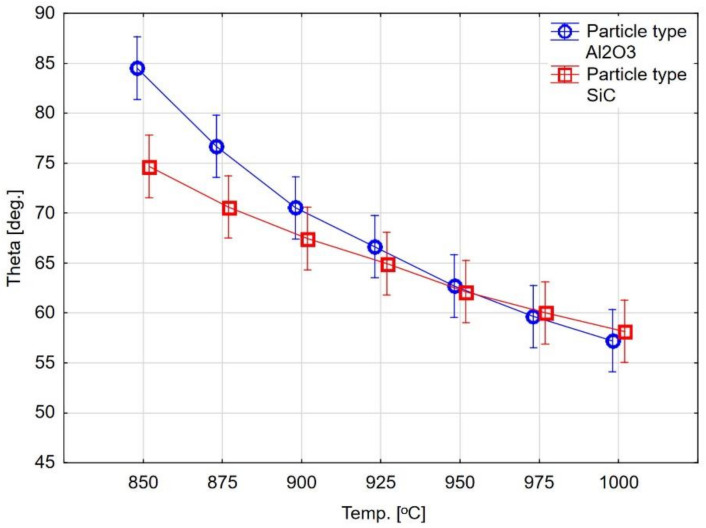
Graph of the dependence of the surface’s contact angles on the temperature for the surface after abrasive blasting with Al_2_O_3_ and SiC.

The surface wettability measurement results are presented in Table 3 and Table 4 and Figure 2 and Figure 3. The research results show the effect of abrasive blasting parameters on ceramics’ contact angles at elevated temperatures. The chart analysis shows that the contact angle decreases with the temperature increase in each case, and thus the wettability of the treated surface by liquid ceramics increases (Figure 3). Moreover, it can be seen that there are differences in substrate wettability trends with temperature change depending on the abrasive blasting parameters (Figure 2).

**Figure 4:**
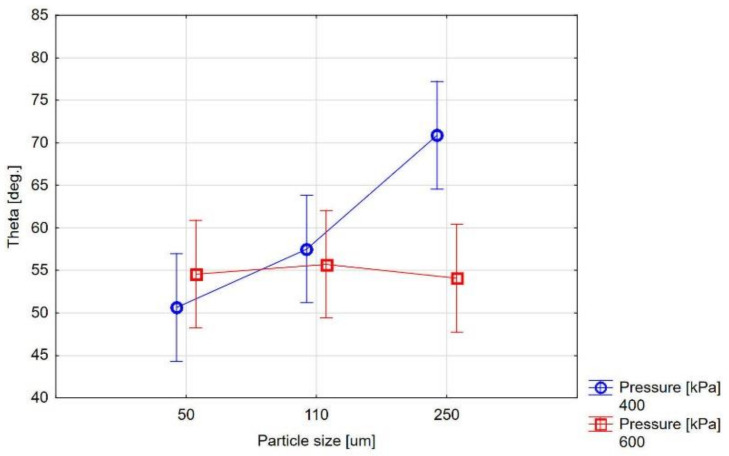
Graph of the dependence of the surface’s contact angles on the particle size and pressure of Al_2_O_3_ abrasive blasting (mean ± 0.95 confidence intervals).

**Figure 5:**
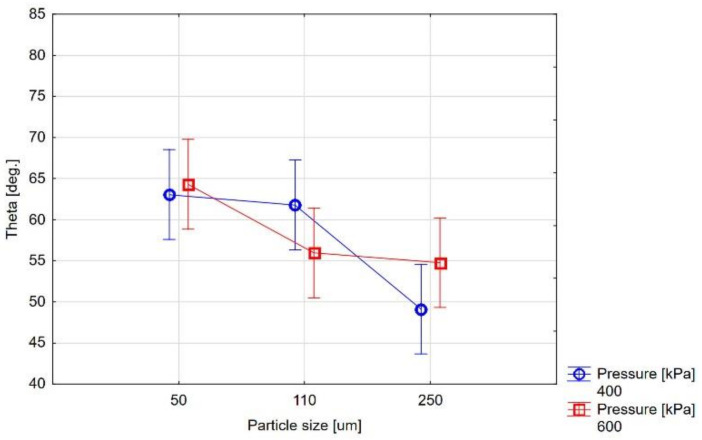
Graph of the dependence of the surface contact angles on the particle size of SiC abrasive blasting (mean ± 0.95 confidence intervals).

The statistical analysis of the measurement results shows that the only important factor affecting the surface’s wettability at temperatures close to firing temperatures is its size of abrasive particles (Figure 4 and Figure 5). In the Al_2_O_3_ abrasive, the abrasive case size correlates with case of treatment pressure, and for larger particle sizes, the higher treatment pressure is beneficial.

The above graphs allow you to determine the temperature at which the liquid ceramics begin to wet the treated surface. The transition temperatures from the non-wetting state to the wetting state are presented in Table 5. From the given data, it can be seen that the obtained contact angle below 90° (θ < 90°—wetting) is achieved for most abrasive blasting parameters below a temperature of 850 °C. The deviating values are visible for Al_2_O_3_ for the large particle at low pressure (400 kPa) and a small particle at high pressure (600 kPa).

The influence of temperature on the relative wetting force, calculating according to Equations (1) and (2), is presented in Figure 5. A significant increase in wettability was observed for large sizes of Al_2_O_3_ abrasive at a pressure of 400 kPa. In the SiC abrasive case, an increase in the wetting force concerning the reference parameters was observed for small abrasive particles.

**Figure 6:**
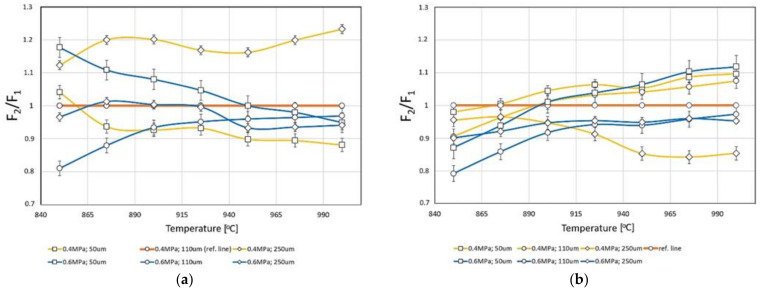
The relative wetting force values for various variants of abrasive blasting concerning the reference treatment (Al_2_O_3_, 400 kPa, 110 µm) depending on the temperature. (**a**) Al_2_O_3_ abrasive, (**b**) SiC abrasive.

The relative wetting force was compared to samples treated with 110 µm abrasive and 400 kPa pressure. This choice was dictated because these parameters were considered the most favorable for the metal-ceramic connection [14,18]. A horizontal line with the value of F_0_/F_1_ represents this treatment variant. Figure 6a shows that for the samples treated with Al_2_O_3_, relative wetting force treated with 50 µm abrasive and 400 kPa is more significant than the reference variant. In other variants of the treatments, the relative wetting force is lower. The situation is slightly different for samples treated with silicon carbide. Samples treated according to variants 50 µm/400 kPa, 110 µm/400 kPa, and 50 µm/600 kPa, from 890 °C, have the relative wetting force more remarkable than the reference sample (110 µm/400 kPa, Al_2_O_3_) in the entire temperature range (Figure 6b).

## 4. Discussion

The research of metal surface wettability with ceramics in prosthetics is scarce, and there are few literature reports on this topic. They mainly concern the study on the wettability of zirconium oxide [17,19,20]. In our tests, Ni-Cr alloy’s wettability was analyzed, which is widely used in the formation of restorations in the form of prosthetic crowns and bridges. The research results show that the metal surface’s wettability with liquid ceramics depends on the abrasive blasting parameters. It is influenced by both the pressure and the type and size of the abrasive used for treatment. The research by Śmielak et al. [17], carried out on zirconium oxide, shows that the wettability also depends on the type of processing (milling, grinding, abrasive blasting). These authors showed that zirconium oxide’s wettability with liquid ceramics increases when the elements are surface treated after shaping (milling). It is related to the change of the material surface condition in relation to the subjected only to milling. The treatment parameters also affect the treated surface condition [15], e.g., its roughness and surface free energy.

Figure 2, Figure 3, Figure 4 and Figure 5 show different contact angles, depending on the abrasive blasting parameters used. As already mentioned, the metal substructure surface’s wettability with liquid ceramics affects this connection’s quality. The optimal treatment, i.e., ensuring the greatest metal–ceramic bond strength, is abrasive blasting with aluminum oxide of 110 μm under a pressure of 400 kPa [11]. The presented research shows that these parameters’ surface wettability is not the highest (Table 3, Figure 2). In principle, the best ceramic wettability can be observed for the surface treated with the smallest (Al_2_O_3_, 50 µm, 400 kPa and Al_2_O_3_, 50 µm, 600 kPa) and largest (Al_2_O_3_, 250 µm, 600 kPa) abrasive particles (Table 3). This is seen in Figure 5, and shows the relative wetting force, which is also greater for these parameters. It can also be seen that the relative wetting force does not reach the highest value for the parameters that, according to literature reports, are optimal for the strength of the metal–ceramic connection. Therefore, it should be assumed that the strength of the connection, apart from wettability, is influenced by other factors. One of these factors may be the amount of embedded abrasive in the metal surface. Research has shown that different amounts of abrasive material are embedded in treated surfaces [15,21,22]. It is not easy to define this impact clearly. On the one hand, the embedded sharp-edged abrasive particles may be the points of fracture in the ceramic, which may contribute to a reduction in strength, and, on the other hand, the dissolution of alumina by liquid ceramics may increase in strength. However, these are only assumptions, and further research is needed to confirm this.

By analyzing the relative wettability force, it can be assumed that the bond strength after treatment with silicon carbide should be better. However, as previously noted, this is not the only factor. In prosthetics silicon carbide is not used for processing. There are no reports on the strength of the metal–ceramic connection after such treatment, so these results cannot be compared to those in the case of aluminum oxide treatments.

The abrasive type’s effect, the dependence of the samples’ wettability after treatment with aluminum oxide and silicon carbide, and 600 kPa pressure are similar for 110 and 250 µm particles. Although the contact angles for the silicon carbide treatment are slightly more significant, the differences between the angles are slight. The wettability for 50 µm particle processing is somewhat different, and the chart’s different nature is visible. The change in contact angle for aluminum oxide is much greater in the same temperature range: from about 98° at 850 °C to about 54° at 1000 °C, while for silicon carbide in the same temperature range, it is from about 72° to about 64°. As for the 400 kPa pressure treatment, the nature of the wettability changes and the temperature change is similar. Still, for silicon carbide, the contact angles are smaller, and the nature of the changes also depends on the particle size. Additionally, in this case, the nature of the curve for the 50 µm abrasive differs from the other two particle sizes. Considering that with the same particle, the surface roughness should be similar, it should rather not be associated with the surface roughness after treatment. Perhaps it is related to the amount of embedded abrasive particles, which, for the same particle size does not have to be analogous for different materials. Silicon carbide particle is a harder and brittle material than aluminum oxide; therefore, particles hitting the treated surface are more easily crushed and bounced off. The degree of crushing and rebound is related to the weight of the individual particles and the operating pressure, affecting the incident’s energy abrasive particles. Clarification of this issue requires a more detailed examination of the amount of embedded abrasive particles after treatment. The explanation can also be obtained by modeling the phenomena occurring during abrasive particles’ impact on the treated surface.

In summary, the abrasive blasting parameters influence the Ni-Cr alloy surface’s wettability with ceramics at elevated temperatures. The surface wettability is influenced by both the surface roughness and the amount of embedded abrasive particles. However, it is not clear to what extent the surface roughness affects the contact angles and the amount of embedded abrasive particles. Since there is a dependence of wetting on the abrasive blasting parameters and the type of abrasive, it seems that the ceramic’s firing temperature is not a constant value. It should be selected each time, depending on the type of surface treatment performed, because of the good flow of the ceramic in the surface’s unevenness. Thus, wet the surface of the metal with liquid ceramics is needed for a good connection.

In practice, the firing of dental ceramics on metal substructure occurs at temperatures in the range of 920 °C. You can see that these are not the temperatures at which the wettability is the best. The presented research shows that with increasing temperature, the contact angles’ values decrease, and the sample surface’s wettability with liquid ceramics increases. Considering only the wettability, the firing temperatures of the ceramics should be higher. Firing temperature restrictions apply. It should be taken into account that the quality of prosthetic restoration is influenced by other factors that may have a negative impact, along with the increase of the firing temperature (change of the alloy structure, high-temperature corrosion, etc.). The temperatures used seem to be optimal. The θ wettability angle is less than 90° at 880 °C for aluminum oxide treatment and 850 °C for silicon carbide treatment. Therefore, it can be seen that, at practically relevant firing temperatures, the liquid ceramic wets the alloy’s surface (angle θ much less than 90°). The obtained results concern only Ni-Cr alloy. They cannot be transferred to other materials. It can be seen from literature reports that, for example, for zirconium oxide at these temperatures, there was no surface wettability because the contact angles were higher than 90 ° [17]. This means that the metal surface has a better surface wetting ability than liquid ceramics compared to zirconium oxide.

## 5. Conclusions

The research shows that the abrasive blasting parameters, like treatment pressure and size of abrasive particles, impact the metal surface wettability at high temperatures. Most likely, it is related to the roughness of tested surfaces or the percentage of embedded abrasive particles. However, there is a significant correlation between the wettability and the size of the used abrasive particles. The wettability of the alloy surface with liquid ceramics increases with increasing temperature and each tested abrasive blasting parameter provides good alloy surface wettability (θ < 90°).

## Figures and Tables

**Table 1:** Chemical composition of the Heraenium^®^ NA alloy (wt.%).

Ni	Cr	Mo	Fe	Mn	Ta	Si	Co	Nb
residue	24.63	9.21	1.53	0.42	0.19	1.54	0.15	0.48

**Table 2:** Designations of the samples according to the parameters of abrasive blasting processes.

Type of Abrasive	Abrasive Particle Size [µm]	Processing Pressure [kPa]	
		400	600
Al_2_O_3_	50	A54	A56
	110	A14	A16
	250	A24	A26
SiC	50	S54	S65
	110	S14	S16
	250	S24	S26

**Table 3:** Wetting angle (degrees) depending on Al_2_O_3_ abrasive blasting parameters and temperature (mean ± standard error).

Temp. [°C]	Al_2_O_3_ Abrasive Blasting Parameters						
	400 kPa			600 kPa			Total
	50 µm	110 µm	250 µm	50 µm	110 µm	250 µm	
**850**	86.33 ± 3.51	82.93 ± 6.83	93.10 ± 2.55	97.56 ± 7.66	67.07 ± 0.81	80.05 ± 3.04	84.51 ± 4.07
**875**	70.20 ± 3.40	74.98 ± 5.63	89.98 ± 2.20	83.10 ± 6.22	65.57 ± 1.33	75.90 ± 2.45	76.62 ± 3.54
**900**	63.80 ± 4.61	68.88 ± 1.41	82.73 ± 3.11	74.38 ± 5.34	64.30 ± 1.66	69.27 ± 1.90	70.56 ± 3.00
**925**	60.23 ± 2.83	65.60 ± 1.72	76.68 ± 1.43	68.65 ± 1.17	62.40 ± 1.14	64.17 ± 4.72	66.29 ± 2.17
**950**	56.75 ± 3.03	63.20 ± 1.92	73.43 ± 1.82	63.18 ± 2.25	60.65 ± 1.42	58.93 ± 5.26	62.69 ± 2.62
**975**	53.58 ± 2.49	59.93 ± 0.75	71.85 ± 1.43	58.70 ± 2.75	57.78 ± 1.49	56.00 ± 5.91	59.64 ± 5.91
**1000**	50.63 ± 2.40	57.50 ± 0.51	70.88 ± 0.83	54.55 ± 2.66	55.73 ± 1.69	54.08 ± 6.14	57.23 ± 6.14
**Total**	63.07± 3.18	67.57 ± 2.68	79.80 ± 1.91	71.45 ± 4.01	61.93 ± 1.36	65.48 ± 4.20	-
ANOVA	Factor			F	P	Partial eta2	Power
	Particle size			5.462	<0.01	0.3777	0.7806
	Pressure × Particle size			6.332	<0.01	0.4130	0.8409

**Table 4:** Wetting angle (degrees) depending on SiC abrasive blasting parameters and temperature (mean ± standard error).

Temp. [°C]	SiC Abrasive Blasting Parameters						
	400 kPa			600 kPa			Total
	50 µm	110 µm	250 µm	50 µm	110 µm	250 µm	
**850**	81.25 ± 2.62	74.67 ± 0.86	79.23 ± 5.40	72.23 ± 0.49	65.57 ± 0.86	74.68 ± 3.64	74.60 ± 2.31
**875**	75.30 ± 1.24	72.20 ± 2.15	72.38 ± 3.98	70.35 ± 1.00	64.38 ± 0.73	69.05 ± 3.06	70.61 ± 2.03
**900**	71.95 ± 0.60	69.58 ± 2.87	65.23 ± 3.49	69.60 ± 0.61	63.15 ± 1.41	65.20 ± 1.25	67.45 ± 1.70
**925**	69.73 ± 0.52	67.70 ± 1.04	59.85 ± 0.74	68.13 ± 1.46	61.75 ± 1.50	62.55 ± 0.46	64.95 ± 0.95
**950**	66.55 ± 0.36	65.80 ± 1.08	53.90 ± 3.01	67.18 ± 1.52	59.35 ± 0.65	59.95 ± 1.48	62.12 ± 1.35
**975**	65.10 ± 0.78	63.35 ± 4.21	50.48 ± 2.20	66.13 ± 1.73	57.40 ± 1.00	57.53 ± 1.44	60.00 ± 1.89
**1000**	63.05 ± 1.01	61.80 ± 4.33	49.10 ± 4.06	64.30 ± 1.63	55.87 ± 0.71	54.78 ± 1.10	58.15 ± 2.14
Total	70.42 ± 1.02	67.87 ± 2.36	61.45 ± 3.27	68.27 ± 1.20	61.07 ± 0.98	63.39 ± 1.78	-
ANOVA	Factor			F	P	Partial eta2	Power
	Particle size			10.325	<0.01	0.5343	0.9694

**Table 5:** Transition temperatures from non-wetted to the wettable state for individual surface samples.

Type of Abrasive	Particle Size [µm]	Treatment Pressure [kPa]	
		400	600
**Al_2_O_3_**	50	<850	863 °C
	110	<850 °C	<850 °C
	250	875 °C	<850 °C
**SiC**	50	<850 °C	<850 °C
	110	<850 °C	<850 °C
	250	<850 °C	<850 °C

## Data Availability

The data presented in this study are available on request from the corresponding author.
